# From the Autochthonous Grape Varieties of the Kastav Region (Croatia) to the Belica Wine

**DOI:** 10.17113/ftb.60.01.22.7264

**Published:** 2022-03

**Authors:** Tomislav Pavlešić, Lara Saftić Martinović, Željka Peršurić, Edi Maletić, Maja Žulj Mihaljević, Domagoj Stupić, Željko Andabaka, Zoran Grgić, Sandra Kraljević Pavelić

**Affiliations:** 1 University of Rijeka, Faculty of Health Studies, Viktora Cara Emina 5, 51000 Rijeka, Croatia; 2 University of Rijeka, Trg braće Mažuranića 10, 51000 Rijeka, Croatia; 3 University of Rijeka, Department of Biotechnology, Radmile Matejčić 2, 51000 Rijeka, Croatia; 4 University of Zagreb, Faculty of Chemical Engineering and Technology, Trg Marka Marulića 19, 10000 Zagreb, Croatia; 5 University of Zagreb, Faculty of Agriculture, Svetošimunska 25, 10000 Zagreb, Croatia

**Keywords:** Belica wine, autochthonous wines, autochthonous grape varieties, polyphenols, FTIR

## Abstract

**Research background:**

Coastal region of Croatia is rich in autochthonous grape varieties. Many of them have been almost abandoned, such as the autochthonous varieties of Kastav (Croatia), used for the production of the Kastavska Belica wine. Therefore, the rationale of the presented study is to characterize autochthonous grape varieties Verdić, Mejsko belo, Jarbola, Divjaka and Brajkovac. In addition, we performed a molecular characterization of the corresponding Belica wines.

**Experimental approach:**

Firstly, the genetic origin and ampelographic and economic characteristics of five autochthonous grape varieties were determined. Standard physicochemical profiles and phenolic components of 12 wines from different producers were determined by liquid chromatography coupled to triple quadrupole mass spectrometer (LC-QQQ-MS). Fourier-transform infrared spectroscopy (FTIR) was used for determination of standard physicochemical parameters.

**Results and conclusions:**

Ampelographic analysis, which includes the data on producing characteristics and cluster and berry composition of the varieties, revealed significant differences between the analysed grape varieties. Results of the physicochemical analysis of the Belica wine showed that all wines met the requirements needed for the production of quality and top quality wines labelled with protected designation of origin (PDO) in Croatian coastal region. The LC-QQQ-MS analysis confirmed the presence of different phenolic components in the Belica wines, where the most prominent phenols were flavonoids from the flavan-3-ol group. Overall, these results showed that autochthonous grapes from the Kastav region can be used for production of wines with added market value due to a growing demand for autochthonous products on the global market.

**Novelty and scientific contribution:**

The presented results give scientific insight and a basis for further determination of the optimal cultivation technology aimed to take advantage of the best characteristics of each variety for production of a wine with desirable features.

## INTRODUCTION

A significant number of grape varieties deserve revitalization, due to their varietal characteristics that may also include resilience in the context of climate changes. In addition, the global market recognizes typical and autochthonous products, such as wines of indigenous grape varieties, often through high prices. In particular, EU has recognized the importance of traditional product sector, not only as a way to strengthen the local economy, but also as a way to generally develop a sustainability system (*1*).

Croatia is rich in indigenous grape varieties. Unfortunately, many of them are still neglected or scientifically uncharacterized. Successful example of a revitalized, nearly forgotten variety is Tribidrag (syn. Crljenak kaštelanski/Primitivo/Zinfandel/Kratošija), of which only 22 vines were found (*2*) near Kaštela. After revelation of its true identity, links to Plavac mali and eastern Adriatic origin (*2*), the renaissance and the resurgence of its production in Croatia began and increased demand for its planting material has been documented (*3*).

Malvazija istarska, Mejsko belo, Divjaka and Jarbola are also unique varieties, grown exclusively in Croatia, whilst synonyms of Verdić are spread over a wider area of the Northern Adriatic coast (*4*) under the names of Teran bijeli (*5*), Glera, Prosecco (*6*) and Beli Teran (*7*) in Slovenia and Prosecco tondo (*8*) in Italy. Apart from the vineyards, they are also conserved in the National Collection of Autochthonous Varieties at the University of Zagreb, Faculty of Agriculture, Croatia. However, that is not the case with Brajkovac, a variety mentioned back in 1853 (*9*), whose varietal status in terms of its uniqueness and possible synonyms/homonyms has not been evaluated before.

With these background data as a rationale for our study, we set the unique interdisciplinary approach for characterization of the autochthonous grape varieties and the corresponding wine. We tested our experimental approach to characterization of the autochthonous varieties of the Kastav region and corresponding Belica wine. Belica wine is a mixture of Mejsko belo, Verdić, Divjaka, Jarbola and Brajkovac grape varieties and belongs to the group of wine made from neglected and somewhat endangered varieties. Some of the varieties have indeed been on the verge of extinction. The Brajkovac variety occurs sporadically in some older vineyards and is used in a small percentage in Belica wine. The Jarbola variety is also present in small amounts. Varieties Divjaka and Mejsko belo are very important for the production of Belica wine and can be found exclusively in the vineyards of the Kastav region.

In this interdisciplinary approach, the ampelographic and economic analysis of varieties that can usually be found in the Belica wine, genetic background of the tested varieties and molecular components of the corresponding Belica wine will be determined. Standard wine chemical parameters are evaluated as well. The Fourier-transform infrared spectroscopy (FTIR) analysis was done as a confirmation of the results along with major phenolic compound evaluation by liquid chromatography coupled to triple-quadrupole mass spectrometry (LC-QQQ-MS).

## MATERIALS AND METHODS

### Samples

**Fig. S1:**
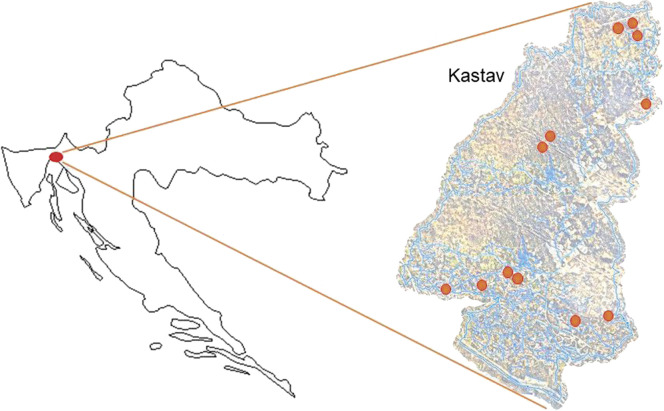
Map of the Kastav area (Croatia). Red circles indicate the locations of the vineyards from which Belica grapes were collected

The ampelographic and genetic research was conducted in the autumn of 2017 at the time of harvest. For wine analysis, total of 12 Belica wine samples (year 2017) were obtained from the local producers of the Kastav area (Croatia) (Fig. S1). The total number of vines ranged from 108–2050 pieces per vineyard of an individual producer. All producers use selected yeast (EC 1118, *Saccharomyces cerevisiae*) for the Belica wine production. The total area of the vineyards and the number of vines on which these twelve producers produce grapes for Belica wine is 13 442 m^2^ and 7285 vine pieces, respectively. The total annual production of Belica wine from these twelve producers is 6470 L. The predominant grape growing system (cultivation form) is single-legged or double-legged Guyot and the substrates are Kober 5 BB and SO4. The maximum yield per hectare for quality wines is 12 000 kg or 8400 L of wine per hectare, or for premium wines 11 000 kg or 6600 L of wine per hectare. Since each producer has different amounts of varieties in the vineyard and no standard guideline has been agreed on so far, it was not possible to determine the exact amount of each variety in the Belica wine.

### Ampelographic and economic analyses of the varieties

Ampelographic analysis includes many parameters for grape variety characterization (morphology, phenology, production characteristic, *etc*.). This study included only parameters important for the production characteristics of the varieties. During the harvest, five healthy and vigorous vines were selected. Total yield and number of clusters were measured by picking, numbering and weighing the clusters from each vine. From the total mass of grapes, ten clusters were randomly sampled for further ampelographic analysis of cluster and berry composition. Cluster composition analysis encompassed measuring dimensions and mass of cluster according to Maletić *et al.* (*10*) and basic chemical composition of must. Berry composition parameters were chosen according to Rustioni *et al*. (*11*). A total of thirteen samples of berries were included for each variety. Berry composition analysis encompassed measurement of dimensions and mass of berry, and skin and seed mass. Skin and seeds of each sample were crushed, placed on paper, dried for two weeks at room temperature and then weighed.

The dimensions of clusters and berries were measured on graph paper. The mass of clusters and berries was measured with a precision laboratory balance (PS 4500.R2.M; Radwag, Radom, Poland). The basic chemical analysis of must comprised the analysis of sugar content (Brix scale, 2352 MASTER-53T refractometer; Atago, Tokyo, Japan) according to OIV-MA-AS2-02 method (*12*), total acid concentration (g/L expressed as tartaric acid equivalents) according to OIV-MA-AS313-01 method (*13*) and pH value (Lab 850 pH meter; Schott Instruments, Mainz, Germany) according to OIV-MA-AS313-15 method (*14*). These three parameters represent the most important quality parameters in wine production. Data obtained by weighing berries and their parts were used to calculate the mass of flesh and mass fraction of skin and seeds in berry composition. Parameter of yield per vine was used to calculate the economic value of grape production. First, grape production was expressed in yield per hectare and multiplied with average price for one kilogram of grapes. Economic analysis was expressed in total revenue, which included variable costs and gross margin.

### Genetic analysis

For variety identification and confirmation, eight vines were sampled. DNA was extracted using the peqGOLD Plant DNA mini kit (PEQLAB Biotechnologie GmbH, Erlangen, Germany) according to the manufacturer's instructions. Nine microsatellite (simple sequence repeats, SSR) primers recommended for routine variety distinction of grapevine were used (*15*). Polymerase chain reaction (PCR) amplifications were carried out in an Applied Biosystems Veriti™ thermal cycler (Thermo Fisher Scientific, Foster City, CA, USA). The list and information about the used primers, as well as detailed information on multiplex PCR reactions performed, are described by Žulj Mihaljević *et al.* (*4*). Amplified products were separated using an Applied Biosystems 3130 genetic analyzer (Thermo Fisher Scientific) with GeneScan™ 500 LIZ^®^ size standard. Sizing of the fragments was performed using GeneMapper v. 4.0 software (*15*). The obtained SSR profiles were compared to internal microsatellite database comprising profiles on 9 common loci from European *Vitis* database (*16*) as well as published SSR profiles from other research (*4*). Data were standardized and compared as described previously (*4*).

### Reagents and materials used in wine analysis

Sodium potassium l(+)-tartrate tetrahydrate was obtained from VWR Chemicals (Vienna, Austria). Potassium iodide was obtained from BDH Prolabo Chemicals (Leuven, Belgium). Iodine, sodium hydroxide (1 and 0.1 M), sodium thiosulfate (0.1 M) and sodium hydroxide pellets were obtained from Gram-mol (Zagreb, Croatia). Sulphuric acid (96%), starch (p.a., soluble) and phenolphthalein were obtained from Kemika (Zagreb, Croatia). Bromothymol blue was obtained from Merck (Darmstadt, Germany). (+)-Catechin, (-)-epicatechin, 2,5-dihydroxybenzoic acid (2,5-DHBA), 3,4-dihydroxybenzoic acid (3,4-DHBA), 3-hydroxytyrosol, caffeic acid, ellagic acid, quercetin, naringenin, luteolin-7-O-glucoside, pinobanksin, *p*-coumaric acid and syringic acid were obtained from Sigma-Aldrich, Merck (St. Louis, MO, USA). Gallic acid was obtained from Alfa Aesar (Thermo Fischer Scientific, Tewksbury, MA, USA). Ferulic acid and resveratrol were obtained from Extrasynthese (Genay, France). Honeywell research chemicals (Charlotte, NC, USA) supplied ethanol (HPLC grade) and acetonitrile (LC-MS grade). Sigma-Aldrich supplied formic acid (LC-MS grade), ultrapure water (LC-MS grade) and ethanol (96%).

### Standard wine analysis

Standard chemical parameters determined for the Belica wine samples were as follows: alcohol, reducing sugar and ash content, pH, total titratable and volatile acidity, and free and total sulphur dioxide content. Total alcohol content was determined by use of the electric ebulliometer (Exacta+Optech Labcenter S.p.A., San Prospero, Modena, Italy) (*17*). For the determination of pH according to OIV-MA-AS313-15 method (*14*), a pH meter Lab 860 (SI Analytics GmbH, Mainz, Germany) was used. Total acidity in the samples was determined according to modified OIV-MA-AS313-01 method (*13*). The only modification in the method was the use of the ultrasonic bath for the elimination of carbon dioxide from the wine instead of a vacuum flask and a water pump. The results were expressed as tartaric acid equivalents. The determination of reducing sugar content in wine samples was carried out according to the method developed by Rebelein (*18*). This is shortened iodometric method based on the titration of iodine formed in the reaction of potassium iodide and unused copper cation (left after the reaction of reducing sugars with alkaline copper sulphate) with sodium thiosulphate. The volatile acid in the samples (expressed as acetic acid equivalents) was determined according to the modified OIV-MA-AS313-02 method (*19*). The only modification in the method was the use of the ultrasonic bath for the elimination of carbon dioxide from the wine instead of a vacuum flask and a water pump. The ash in the wine samples was determined according to OIV-MA-AS2-04 method (*20*). Free and total sulfur dioxide were determined by the titration with a standard solution of iodine. Methods were developed according to OIV-MA-AS323-04B method (*21*) and the rapid method by Ripper (*22*). For free SO_2_ determination, 5 mL of diluted H_2_SO_4_ (1:3) with 2 mL of 1% starch were added to 50 mL of the sample and titrated with 0.01 M solution of J_2_ until a blue colour appeared. The consumption of J_2_ was multiplied by a factor of 12.8 and the results were expressed in mg/L of free SO_2_ in the sample. For total SO_2_ determination, 25 mL of 1 M NaOH were added to 50 mL sample and left to stand for 15 min. Afterwards, 10 mL of diluted H_2_SO_4_ (1:3) and 2 mL of 1% starch were added and titrated with 0.01 M J_2_ solution until a blue colour appeared. The titration consumption was multiplied by a factor of 12.8 and the results were expressed in mg/L of total SO_2_ in the sample.

### Fourier-transform infrared spectroscopy analysis

Infrared spectra were recorded with WineScan^TM^ FTIR spectrometer (FOSS, Hillerød, Denmark) within mid-IR (1000-5000 cm^-1^) range. The used samples were directly collected from the bottle without any pretreatment. Calibrations that are part of the WineScan^TM^ FTIR allowed simultaneous analysis of major wine quality parameters such as alcohol, total acidity, volatile acidity and reducing sugars.

### LC-QQQ-MS analysis

Wine samples were diluted twice with 10% ethanol solution, filtered through Chromafil cellulose acetate microfilters (0.45 µm, 25 mm; Macherey-Nagel, Düren, Germany) and analysed. LC-QQQ-MS analysis was performed with Agilent 1260 series HPLC chromatograph equipped with a degasser, binary pump, auto-sampler and column oven coupled to an Agilent 6460 triple quadrupole mass spectrometer equipped with jet stream electrospray (AJS ESI) source (Agilent Technologies, Palo Alto, CA, USA). For chromatographic separation, Zorbax SB-C18, rapid resolution HT, 6·10^7^ Pa column (2.1 mm×50 mm i.d, 1.8 µm; Agilent Technologies) was used. The mobile phases were 0.1% formic acid in LC-MS grade water (A), and 0.1% formic acid in acetonitrile (B). Details of the method used for quantification of flavonoids and phenolic acids are described in our previous publication (*23*). Parameters for calibration curves of the analysed phenolic compounds (linearity, limit of detection (LOD), limit of quantification (LOQ) and coefficient of determination (R^2^) used for quantification of phenolic compounds are given in Table S1.

### Statistical analysis

The obtained data were statistically processed using the SAS software, v. 9.3. (*24*). Statistical analysis included descriptive statistics (average, minimum and maximum value), analysis of variance (one-way ANOVA) and comparison of mean values (Duncan’s multiple-range test). Principal component analysis (PCA) was constructed using Python library Scikit-learn v. 0.20.3 (*25*) was used for both classifiers.

## RESULTS AND DISCUSSION

### Ampelographic characteristics of varieties

All analysed varieties are white skin grapes with specific morphological characteristics. The results of one-way ANOVA of production characteristics showed significant differences in most of the parameters except for cluster mass, skin mass and mass fraction of skin and seeds (Table 1). Verdić had the largest cluster by dimensions (average length (188±23) mm, average width (116±15) mm), while Jarbola had the smallest clusters by dimension (average length (126±22) mm, average width (79±14) mm) (Table 2). Even though cluster mass was not significant, the ANOVA comparison of the mean values showed that a difference between varieties exists. Mejsko belo had the greatest and Jarbola the smallest cluster mass. Our analysis of the berry composition showed that Mejsko belo had the largest berry ((16.6±1.6) mm average length and (14.9±2.0) mm average width), while Jarbola had the smallest one ((13.9±1.3) mm average length and (12.5±1.2) mm average width) (Table 3). Variety Verdić also had the highest values for majority of other parameters: berry mass, flesh, skin and seed mass, and mass fraction of skin. All average values of the analysed varieties are very close to the average values of 22 383 data from the sample analysis in the study of Rustioni *et al*. (*11*). For example, the values of berry length/width ratio for Verdić and Brajkovac are the same as the average in the mentioned study. Varieties Mejsko belo and Brajkovac have the highest value of this parameter, which confirms the characteristic morphological ovoid shape of the berry.

**Table 1:** Results of one-way ANOVA for cluster and berry composition parameters

Parameter	Pr>F(Model)	Significant
*l*(cluster)/mm	<0.0001	Yes
*b*(cluster)/mm	0.004	Yes
*m*(cluster)/g	0.176	No
*l*(berry)/mm	<0.0001	Yes
*b*(berry)/mm	<0.0001	Yes
*l*/*b*	0.005	Yes
*m*(berry)/g	0.005	Yes
*m*(flesh)/g	0.011	Yes
*m*(skin)/g	0.440	No
*m*(seed)/g	0.004	Yes
*w*(skin)/%	0.794	No
*w*(seed)/%	0.075	No

**Table 2:** Results of descriptive statistics (mean values with standard deviation) and comparison of mean values (Duncan’s multiple-range test) for cluster parameters of five autochthonous grape varieties of Kastav region (Croatia)*

**Grape variety**	***l*(cluster)/mm**	***b*(cluster)/mm**	***m*(cluster)/g**
**Verdić**	(188±23)^a^	(116±15)^a^	(262±96)^ab^	
**Mejsko belo**	(164±32)^b^	(113±31)^a^	(280±118)^a^	
**Brajkovac**	(1462±18)^bc^	(104±32)^a^	(233±73)^ab^	
**Divjaka**	(145±20)^bc^	(88±21)^b^	(237±88)^ab^	
**Jarbola**	(126±22)^c^	(79±14)^c^	(184±59)^b^	

*Sample of 10 clusters. Different letters show a statistically significant difference ​​between varieties at p<0.05 (Duncan’s multiple range test)

**Table 3:** Results of descriptive statistics (mean values with standard deviation) and comparison of mean values (Duncan’s multiple-range test) for berry dimension parameters of five autochthonous grape varieties of Kastav region (Croatia)*

**Grape variety**	***l*(berry)/mm**	***b*(berry)/mm**	***l*/*b***	***m*(berry)/g**	***m*(flesh)/g**	***m*(skin)/g**	***m*(seed)/g**	***w*(skin)/%**	***w*(seed)/%**	
**Verdić**	(15.6±1.5)^b^*	(14.76±1.4)^a^	(1.05±0.1)^b^	(63.32±9.1)^a^	(59.21±9.6)^a^	(2.67±0.8)^a^	(1.43±0.2)^a^	(4.34±1.6)^a^	(2.30±0.5)^ab^	
**Mejsko belo**	(16.61±1.6)^a^	(14.94±1.9)^a^	(1.12±0.1)^a^	(62.09±2.3)^a^	(58.88±1.8)^a^	(2.14±0.9)^a^	(1.06±0.1)^b^	(3.42±1.4)^a^	(1.72±0.2)^ab^	
**Brajkovac**	(13.96±1.5)^d^	(13.16±1.3)^b^	(1.06±0.1)^b^	(56.05±1.7)^ab^	(52.74±2.2)^ab^	(1.91±0.3)^a^	(1.39±0.3)^ab^	(3.42±0.6)^a^	(2.49±0.6)^a^	
**Divjaka**	(14.71±1.4)^c^	(13.52±1.6)^b^	(1.09±0.1)^ab^	(48.84±4.9)^b^	(46.19±4.8)^b^	(1.57±0.5)^a^	(1.08±0.1)^b^	(3.22±1.1)^a^	(2.23±0.3)^ab^	
**Jarbola**	(13.89±1.3)^d^	(12.51±1.2)^c^	(1.11±0.1)^a^	(47.07±0.3)^b^	(44.42±0.7)^b^	(1.93±0.7)^a^	(0.71±0.2)^c^	(4.11±1.6)^a^	(1.51±0.4)^b^	

*Sample of thirteen berry. Different letters show a statistically significant difference ​​between varieties at p<0.05 (Duncan’s multiple range test)

Considering the results of the analysed production characteristics of varieties, these also varied (Table 4). According to the cluster and berry analyses, Verdić showed to be the variety of large cluster, due to lower load (ten bunches per vine) and yield per vine (2.66 kg), and had the best quality of must. Additionally, both Verdić and Mejsko belo varieties had the highest sugar content (17.4 °Brix), and thus can be confirmed as the varieties with the best qualitative potential. Total acid concentration is very important quality parameter because it affects biochemical stability and organoleptic character of wine. Low acid concentration in must (3 to 5 g/L, like in Verdić and Mejsko belo) is not favourable from the technological aspect as it indicates artificial acidification of wine.

**Table 4:** Production and economical characteristics of five autochthonous grape varieties of the Kastav region (Croatia)

Grape variety	*N̅*(bunch)	Total sugar/°Brix	*γ*(total acids)/(g/L)	pH	*Y̅*/kg	Average price/(€/kg)	Revenue/(€/ha)	Variable costs/(€/ha)	Gross margin/(€/ha)
Divjaka	15.0	13.8	8.05	3.04	3.36	1.20	23 889.60	5606.36	18 283.24
Jarbola	12.2	15.8	5.27	3.05	2.47	0.93	13 610.32	5150.84	8003.96
Mejsko belo	12.2	17.4	3.51	3.22	3.14	0.66	12 278.97	5493.76	6672.61
Verdić	10.0	17.4	4.19	3.13	2.66	0.73	11 505.17	5248.08	5898.81
Brajkovac	16.0	15.2	5.86	3.15	3.56	1.20	25 311.60	5708.72	19 705.24

**Fig. 1:**
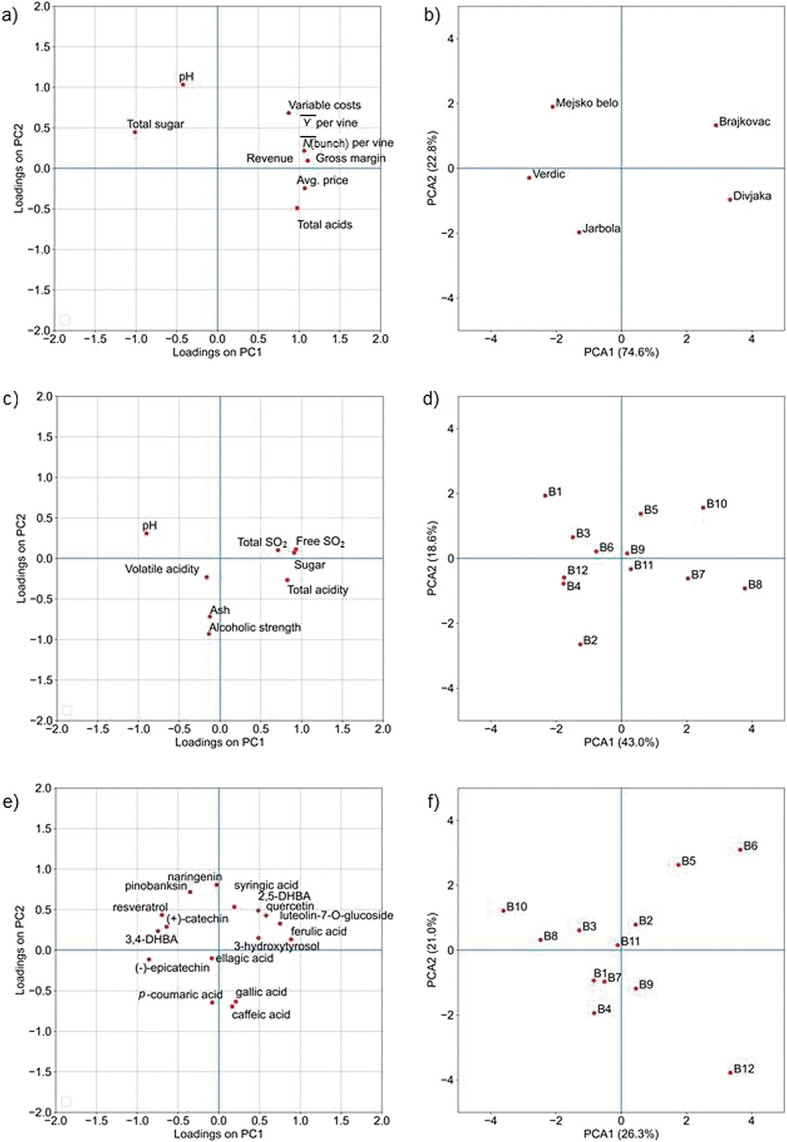
Distribution of elements (variables and samples) in the space of principal component 1 (PC1) and principal component 2 (PC2) when used as variables: a and b) standard physicochemical parameters of grape varieties, c and d) standard physicochemical parameters of Belica wines, e and f) phenolic compounds in Belica wines

Varieties Brajkovac and Divjaka in the observed year had the highest number of clusters and the highest yield per vine, over 3 kg. Such a high yield also affected the quality of the must, so both varieties had the lowest sugar content and the highest concentration of total acids (Table 4). On the other hand, high concentration of total acids, especially in Divjaka had a great variety potential for natural correction of acid concentration in Belica wine as a variety blend wine. In order to test the potential grouping of the samples and get deep insight into the differences between Belica wines, we performed PCA analysis (Fig. 1). Figs. 1a and 1b show the PCA projection of all analysed varieties and their potential grouping in the space of major components. The first two components (PC1 and PC2) describe 97.4% of the total variability. These results confirm a large difference between grape varieties in terms of standard chemical parameters.

Overall, from a technological point of view, in the future more attention should be paid to the reduction of yields of all varieties in order to achieve a higher concentration of sugar in the must but keep optimal acid concentration. Yield reduction in all varieties should be achieved by a combination of stronger pruning to maturity (leaving a smaller number of buds) and subsequent thinning of the clusters (after flowering or before the beginning of the ripening of clusters) and early defoliation. The results showed better insight into the agrobiological properties of these varieties, unexplored so far. However, we need to emphasize that this is a one-year study, and that results can differ depending on the year of production. Therefore, for the final confirmation of the obtained results, a multi-year research should be carried out under the above environmental conditions. The analysed varieties differ in their production characteristics (yields per vine and hectare), but also in economic characteristics (sale price, revenue and gross margin). The relationship between yield and selling price shows the highest profitability of growing varieties Brajkovac and Divjaka. Despite the relatively high yield of the variety Mejsko belo, it achieves lower revenues and coverage contribution due to the lowest selling price.

It can be assumed that the market design (branding) of Kastav Belica wine will contribute to the increase of the total income of wines of a mixture of autochthonous varieties, as well as individual varietiy wines.

### Results of genetic analysis

Belica is a blend (cuvée or mixture) of several grape varieties. The following grape varieties can be found in Belica, most of which are found only in the Kastav region: Mejsko belo, Verdić, Divjaka, Jarbola, Malvazija istarska and Brajkovac. Among them, four (Mejsko belo, Verdić, Malvazija istarska and Divjaka) are the most represented. Malvazija istarska was not analysed in this paper, as it is not autochthonous variety of Kastav area, but of Istrian peninsula. All eight accessions were successfully amplified (Table 5). As expected, true-to-type nature of Mejsko belo, Verdić, Divjaka and Jarbola was confirmed after comparison with internal, previously harmonized SSR database (*4*) containing more than 2000 nonredundant grape genotypes. Four vines were sampled and assumed under the name Brajkovac; however, three different genetic profiles were obtained. Accessions labelled BRAJ_ORIG1 and BRAJ_ORIG2 showed to be identical to Duranija and Mejsko belo, respectively. Nevertheless, two accessions (BRAJ_A and BRAJ_B) showed identical profiles on all nine analysed SSR markers. The obtained genetic profile did not match any other previously known variety/genotype from the SSR database nor has this name appeared in foreign literature, thus confirming the unique status of this variety. These two accessions were further considered as true Brajkovac and were subject of further analysis. This result enables next conservation steps needed for the preservation of this variety, like its inclusion in National collection of autochthonous grapevine varieties settled at the Faculty of Agriculture in Zagreb, Croatia. Also, financial support of local authorities for propagation of the planting material will be beneficial for producers that will accordingly be able to plant those varieties in future.

**Table 5:** Microsatellite profiles and their genetic match on 9 SSR loci for eight analysed accessions. Alleles are presented as base pairs

Accession name	VVS2		VVMD7		MD27		VrZAG62		VrZAG79		VVMD5		VVMD25		VVMD28		VVMD32		Match
BRAJ_A*	141	151	245	261	175	177	193	203	234	256	224	232	253	261	232	276	272	272	BRAJ_A=BRAJ_B
BRAJ_B*	141	151	245	261	175	177	193	203	234	256	224	232	253	261	232	276	272	272	BRAJ_A=BRAJ_B
BRAJ_ORIG1*	131	143	237	245	175	177	187	203	234	256	222	224	237	239	256	276	272	272	Duranija
BRAJ_ORIG2*	141	143	237	245	175	177	187	193	234	256	222	224	239	253	247	256	272	272	Mejsko belo
Mejsko belo	141	143	237	245	175	177	187	193	234	256	222	224	239	253	247	256	272	272	Mejsko belo
Divjaka	141	149	245	261	175	177	193	203	234	256	224	232	253	261	232	276	272	272	Divjaka
Verdić	131	141	237	245	175	190	187	203	246	256	222	242	237	241	234	242	262	264	Verdić
Jarbola	141	153	245	247	175	177	201	203	240	256	232	237	241	253	234	244	250	272	Jarbola

*The autochthonous grape variety Brajkovac samples

### Belica wine analysis

According to physicochemical parameters, all analysed Belica wines meet the requirements for quality wine produced under the label protected designation of origin (PDO) on the Croatian coast (Table 6). FTIR analysis confirmed these results (Table S2). Particularly, the alcohol volume fractions in Belica wine samples were between 11.4 and 13.5% (Table 6). As for the sugar concentration, the results show that all the analysed samples are within the limits of dry wine production (1.05-3.89 g/L). Ash concentration was in the range 0.8 to 1.9 g/L. According to the regulation established for PDO Croatian coastal region (*26*), the minimum concentration of ash for white wines in the category of quality wine with controlled geographical origin is 1.4 g/L. From the obtained results, it is evident that all except one (sample B10) analysed wine samples meet the given criteria. Results of the analysis of total acids in wine showed that all the analysed samples are above the minimum legal limit of wine production (3.5 g/L), whereas the lowest measured concentration of total acids in Belica wine samples was 4.50 g/L. Furthermore, the upper limit for the volatile acid concentration was 1.1 g/L. It is evident that all analysed wines meet the given criteria. Results of the determination of the free sulfur in wine showed that in samples B1 to B4, B6 and B12, the measured concentration was too low, and the wines were in a state of oxidation. After the analysis of total sulfur in wine, all relevant samples of Belica wine complied with the regulation on wine, whereas the upper limit for white and rosé wines is 200 mg/L of free SO_2_ (Table 6). The PCA projection of the standard physicochemical parameters (Figs. 1c and 1d) shows moderate variability among Belica wine samples. The first two components (PC1 and PC2) describe 61.6% of the total variability. These results show that a more uniform production of Belica wine should be pursued.

**Table 6:** Results of standard analyses of Belica wine samples from the Kastav region (Croatia)

Wine sample	*φ*(alcohol)/%	*γ*(sugar)/(g/L)	*γ*(ash)/(g/L)	pH	*γ*(acidity)_total_/(g/L)	*γ*(acidity)_volatile_/(g/L)	*γ*(SO_2_)_free_/(mg/L)	*γ*(SO_2_)_total_/(mg/L)
B1	11.4	1.30	1.5	3.48	4.50	0.22	5	98
B2	13.5	1.20	1.8	3.13	5.70	0.28	6	66
B3	12.0	1.74	1.6	3.35	5.10	0.20	7	78
B4	12.5	1.09	1.8	3.30	5.77	0.20	4	56
B5	11.7	1.66	1.4	3.15	4.87	0.22	25	141
B6	12.2	1.47	1.5	3.19	5.02	0.25	6	133
B7	12.6	3.89	1.8	3.10	5.92	0.15	20	128
B8	12.1	3.15	1.9	2.97	7.12	0.25	32	190
B9	12.3	2.36	1.4	3.18	5.17	0.30	23	87
B10	11.9	3.06	0.8	2.98	6.82	0.20	20	106
B11	12.9	1.60	1.6	3.19	5.10	0.15	16	170
B12	12.9	1.05	1.5	3.22	4.57	0.24	6	95

During the winemaking process, a number of chemical modifications that occur significantly affect the final phenolic profile of the wine, for example, grape ripeness, processing methods and environmental factors. Therefore, a systematic quantitative analysis of phenolic components in wine can provide reliable data on their quantity and type. During the handling and ripening of the grapes, the composition of the polyphenols in the wine may change. Phenolic analysis is therefore crucial to draw conclusions about the winemaking process, as well as about the final wine quality.

Phenolic content in Belica wines was analysed by LC-QQQ-MS. The results showed that the most common groups of polyphenols are hydroxycinnamic acids and flavan-3-ols, which is in line with previous research on white wines (*27*–*29*). Among all hydroxycinnamic acids, caffeic acid was present at highest concentrations, ranging from (0.8±0.4) to (9.1±0.1) mg/L (Table 7). Lukić *et al*. (*30*) conducted qualitative research of phenols in different white wines and reported different trends. Namely, in their research, the phenolic acid with the highest concentration was the gallic acid, with concentrations up to (16.68±15.30) mg/L in Muscat Blanc wine. Our results report significantly lower concentrations of this phenolic acid in the analysed samples ((0.39±0.00) to (1.9±1.1) mg/L). However, the results of the caffeic and ferulic acid concentrations were similar to the results of Lukić *et al.* (*30*). Rochetti *et al.* (*31*) reported lower concentrations of caffeic acid in Chardonnay wines (up to (0.26±0.15) mg/L), which is more in line with our results. Along with ferulic acid, their results for syringic and ferulic acid concentrations were also in line with our data.

**Table 7:** Mass concentration of specific phenolic acids and flavonoids in Belica wine samples obtained by LC-QQQ-MS method

Wine sample	*γ*/(mg/L)															
2,5-DHBA	3,4-DHBA	caffeic acid	ellagic acid	ferulic acid	gallic acid	*p*-coumaric acid	syringic acid	(+)-catechin	(-)-epicatechin	3-hydroxy tyrosol	quercetin	luteolin-7-*O*- glucoside	naringenin	pinobanksin	resveratrol
B1	0.58±0.05	2.2±0.1	2.4±1.2	2.0±0.1	0.04±0.04	1.0±0.1	0.52±0.01	0.41±0.00	7.8±6.0	2.7±2.1	1.60±0.01	-	-	0.06±0.00	0.15±0.01	0.04±0.00
B2	0.32±0.02	1.0±0.2	0.8±0.4	0.13±0.02	0.05±0.01	0.39±0.00	0.11±0.06	0.40±0.01	9.70±0.03	1.2±0.8	0.49±0.01	0.03±0.00	0.04±0.03	0.05±0.01	0.20±0.01	0.04±0.01
B3	0.43±0.03	2.62±0.02	7.01±0.08	0.23±0.02	0.05±0.01	0.85±0.08	0.69±0.06	0.43±0.03	10.2±0.3	2.0±0.4	1.04±0.08	-	0.02±0.00	0.11±0.01	0.30±0.00	0.05±0.00
B4	0.46±0.01	1.53±0.02	8.21±0.03	0.3±0.2	0.05±0.00	0.64±0.03	0.7±0.5	-	8.6±2.0	3.72±0.01	1.9±0.1	0.01±0.00	0.01±0.00	0.07±0.00	0.19±0.00	0.04±0.00
B5	0.99±0.01	1.18±0.02	1.2±0.8	0.25±0.06	0.11±0.02	0.50±0.03	0.4±.03	0.45±0.00	6.4±4.2	1.4±0.7	1.2±0.2	-	0.04±0.00	0.12±0.01	0.28±0.04	0.04±0.00
B6	0.68±0.00	1.21±0.09	1.79±0.05	0.30±0.09	0.17±0.05	0.50±0.04	0.4±0.3	0.48±0.03	9.0±0.3	1.5±0.4	2.3±0.3	0.20±0.07	0.04±0.00	0.10±0.02	0.22±0.07	0.03±0.00
B7	0.34±0.03	0.73±0.05	4.0±0.2	0.10±0.01	0.10±0.01	0.41±0.01	0.64±0.01	0.2±0.3	11.33±0.04	3.38±0.03	0.6±0.2	-	0.02±0.00	0.05±0.01	0.21±0.02	0.04±0.00
B8	0.14±0.01	3.0±0.1	2.23±0.02	0.05±0.01	0.07±0.00	1.0±0.1	0.40±0.01	0.44±0.06	14.7±0.9	3.9±0.2	0.5±0.1	0.05±0.01	-	0.08±0.00	0.25±0.01	0.03±0.00
B9	0.25±0.01	1.17±0.01	1.29±0.00	0.20±0.07	0.07±0.01	0.45±0.04	0.20±0.02	0.2±0.3	7.9±0.6	1.8±0.5	0.4±0.1	-	0.03±0.00	0.04±0.00	0.10±0.01	0.03±0.01
B10	0.30±0.00	2.27±0.06	0.90±0.05	0.21±0.01	0.03±0.04	0.63±0.03	0.19±0.02	0.41±0.02	13.2±7.7	4.7±0.2	0.32±0.00	-	0.01±0.00	0.07±0.01	0.25±0.01	0.07±0.01
B11	0.21±0.00	1.11±0.01	1.15±0.05	0.02±0.00	0.09±0.02	0.49±0.02	0.49±0.02	0.40±0.00	17.0±2.2	2.8±0.3	0.54±0.05	0.01±0.00	0.04±0.01	0.07±0.00	0.18±0.03	0.02±0.00
B12	0.3±0.2	0.62±0.01	9.1±0.1	0.12±0.01	0.14±0.06	1.9±0.1	0.8±0.2	0.42±0.00	4.4±0.1	1.4±0.1	1.0±0.4	0.04±0.01	0.03±0.00	0.03±0.01	0.12±0.00	0.01±0.00

Results are expressed as a mean value±standard deviation (S.D.), *N*=2

As for flavan-3-ols, catechins were found in the highest concentrations, with (+)catechin being dominant ((4.4±0.1) to (17.0±2.2) mg/L) (Table 7). The catechins are responsible for the bitterness in wine (*32*-*34*) but also for wine health properties (*35*). In addition to sensory properties, they are also important as antioxidants, *i.e.* factors that protect the wine from oxidation during maturation (*36*, *37*). These results are in line with the work by Lukić *et al.* (*30*), who reported concentrations of epicatechin and catechin in white wines in the range from (3.10±2.60) to (17.92±13.10) and from (1.51±1.82) to (3.54±2.99), respectively. Rochetti *et al.* (*31*) reported lower concentrations of catechin in Chardonnay wines, ranging from (1.19±0.49) to (6.81±2.53) mg/L. In addition, we also reported higher hydroxytyrosol content in Belica wines, up to (2.3±0.3) mg/L, while the highest concentration in Chardonnay wines was (0.9±0.2) mg/L.

Figs. 1e and 1f show the PCA projection of LC-QQQ-MS quantitative phenolic analysis where all analysed phenols were used as variables. The first two components describe 47.3% of the total variability. Although most wine samples are grouped centrally, samples B5, B6, B10 and B12 contribute to a larger variability of the system. Large differences may be observed in single polyphenol concentrations among samples, showing a need for the development of a more uniform method of Belica wine production.

## CONCLUSIONS

The tested interdisciplinary approach for characterization of selected autochthonous grape varieties and corresponding wine samples proved useful in the assessment of important parameters for branding and quality assessment. Analyses of ampelographic characteristics of Verdić, Mejsko belo, Jarbola, Divjaka and Brajkovac varieties used for the production of Belica wine in the region of Kastav (Croatia) showed high genetic variability among the confirmed grape varieties. Confirmed variability certainly impacts the productivity and economic aspect of the Belica wine production. Results of this study are crucial in the determination of an optimal cultivation technology as it is required by modern trends in grape and wine production. Microsatellite genetic profile and uniqueness of the new variety Brajkovac was confirmed. The knowledge of standard wine characteristics coupled with molecular analyses may be used to evaluate the best characteristics of each variety and establish the production of a wine with desirable characteristics. Recording and monitoring of typical molecular composition of wine through different years, often called molecular profiling may be an important tool for standardization and/or monitoring of the technological process of the production, protection and branding of this autochthonous Belica wine. Current global market trends, indeed, emphasize local, specific and autochthonous products, increasing their demand. Currently, there are large differences in the production characteristics of tested varieties, which is determined by differences in yield and selling prices. Further work on branding the Kastavska Belica wine might increase this specific wine quality and provide benefits to the producers.

## SUPPLEMENTARY MATERIALS

Supplementary materials are available at www.ftb.com.hr.

## Figures and Tables

**Table S1:** LC-QQQ-MS parameters for phenolic acid and flavonoid analysis of Belica wine samples

**Compound**	***t*/min**	**Polarity**	***m*/*z* precursor ion**	*m/z* product ion*	**Collision energy/V**	**Y=ax+b**		**Linearity range/(µg/mL)**	**R^2^**	**LOD/(µg/mL)**	**LOQ/(µg/mL)**
Slope (a)	Intercept (b)
**Phenolic acid**											
**2,5-DHBA**	4.42	-	152.8	108.0 81.8 53.0	20 16 20	15806.66	33.47	0.001-7.5	0.995	0.007	0.021
**3,4-DHBA**	1.70	-	152.9	108.0 81.0 53.0	20 16 18	3761.51	15.01	0.005-7.5	0.998	0.013	0.040
**caffeic**	5.35	-	178.8	135.0 116.9 88.9	12 24 34	27277.16	206.79	0.005-5.0	0.991	0.025	0.076
**ellagic**	6.70	-	301.0	283.7 228.4 244.6	28 30 32	898.41	291.00	1.0-5.0	0.997	1.069	3.239
**ferulic**	6.70	-	192.9	177.9 149.0 134.0	8 6 12	2289.69	4.91	0.01-10.0	0.997	0.007	0.021
**gallic**	0.86	-	168.8	125.0 78.9	10 20	11611.28	58.78	0.01-7.5	0.998	0.017	0.051
***p*-coumaric**	6.26	-	162.9	119.0 92.8 64.9	12 36 48	30897.42	769.42	0.025-2.5	0.991	0.082	0.249
**syringic**	5.59	-	197.0	181.8 166.9 122.6	8 16 22	463.54	-89.15	0.25-7.5	0.992	0.6347	1.923
**Flavonoid**											
**(+)-catechin**	5.20	-	298.0	244.8 204.9 122.7 108.8	10 12 30 26	1753.68	7.32	0.1-5.0	0.997	0.014	0.042
**(-)-epicatechin**	5.70	-	298.1	244.9 108.9	10 26	2893.55	-0.61	0.01-2.5	0.999	0.001	0.002
**3-hydroxytyrosol**	1.73	-	152.9	95.0 94.8	18 20	756.90	18.41	0.1-2.5	0.994	0.080	0.243
**quercetin**	9.15	-	300.9	178.8 151.0 120.9	14 18 24	17670.76	-23.67	0.1-1.0	0.992	0.004	0.013
**luteolin-7-O-glucoside**	6.80	+	449.1	287.0	14	122734.19	117.66	0.001-2.5	0.999	0.003	0.010
**naringenin**	10.16	-	270.9	151.0 118.9	12 24	33448.04	10.74	0.001-0.5	0.998	0.001	0.003
**pinobanksin**	10.10	-	271.0	252.9 225.0 196.6 160.7	18 18 24 24	10928.37	27.17	0.01-1.0	0.998	0.008	0.025
**resveratrol**	8.31	+	228.8	163.3 107.2 135.0	26 8 20	7089.69	-3.93	0.001-10.0	0.995	0.002	0.005

*Quantifier ions are underlined. LOD=limit of detection, LOQ=limit of quantification, DHBA=dihydroxybenzoic acid

**Table S2:** Results of the Fourier-transform infrared spectroscopy (FTIR) analysis of Belica wine samples from Kastav region (Croatia)

Wine sample	*φ*(alcohol)/%	*γ*(glucose and fructose)/(g/L)	*γ*(total acidity)/ (g/L)	*γ*(malic acid)/ (g/L)	*γ(*lactic acid)/ (g/L)	*γ*(volatile acidity)/(g/L)	pH	Specific gravity
B1	11.5	2.6	4.7	0.6	1.4	0.20	3.39	0.9915
B2	12.5	2.0	5.9	1.7	0.2	0.25	3.15	0.9907
B3	12.9	1.9	5.6	2.5	0	0.18	3.32	0.991
B4	11.6	1.9	6.1	2.6	0	0.23	3.35	0.9922
B5	12.3	2.5	5.2	1.9	0	0.21	3.25	0.9904
B6	12.3	2.2	5.5	2.2	0	0.23	3.27	0.9909
B7	13.0	4.4	6.3	2.6	0	0.16	3.09	0.9915
B8	11.4	3.3	7.0	2.9	0	0.24	3.15	0.9924
B9	12.9	2.3	5.6	1.6	0	0.29	3.10	0.9913
B10	13.5	3.4	6.7	2.3	0	0.20	3.33	0.9907
B11	11.2	2.0	5.5	2.2	0	0.12	3.48	0.9913
B12	12.3	1.9	5.0	1.7	0	0.18	3.27	0.9908
